# Serum peptidome: diagnostic window into pathogenic processes following occupational exposure to carbon nanomaterials

**DOI:** 10.1186/s12989-021-00431-0

**Published:** 2021-10-28

**Authors:** Ekaterina Mostovenko, Matthew M. Dahm, Mary K. Schubauer-Berigan, Tracy Eye, Aaron Erdely, Tamara L. Young, Matthew J. Campen, Andrew K. Ottens

**Affiliations:** 1 Department of Anatomy and Neurobiology, Virginia Commonwealth University, PO Box 980709, Richmond, VA 23298 USA; 2 Division of Field Studies and Engineering, National Institute for Occupational Safety and Health, 1090 Tusculum Avenue, MS-R12, Cincinnati, OH 45226 USA; 3 Evidence Synthesis and Classification Section, International Agency for Research On Cancer, 150 Cours Albert Thomas, 69372 Lyon, CEDEX 08 France; 4 Health Effects Laboratory Division, National Institute for Occupational Safety and Health, 1095 Willowdale Road, MS-2015, Morgantown, WV 26505 USA; 5 Department of Pharmaceutical Sciences, University of New Mexico, MSC09 53601, Albuquerque, NM 87131 USA

**Keywords:** Carbon nanotubes, Carbon nanofibers, Biomarkers, Peptidomics, Mass spectrometry, Cardiovascular, Health outcomes, Occupational, Nanotoxicology, Nanomaterials

## Abstract

**Background:**

Growing industrial use of carbon nanotubes and nanofibers (CNT/F) warrants consideration of human health outcomes. CNT/F produces pulmonary, cardiovascular, and other toxic effects in animals along with a significant release of bioactive peptides into the circulation, the augmented serum peptidome. While epidemiology among CNT/F workers reports on few acute symptoms, there remains concern over sub-clinical CNT/F effects that may prime for chronic disease, necessitating sensitive health outcome diagnostic markers for longitudinal follow-up.

**Methods:**

Here, the serum peptidome was assessed for its biomarker potential in detecting sub-symptomatic pathobiology among CNT/F workers using label-free data-independent mass spectrometry. Studies employed a stratified design between High (> 0.5 µg/m^3^) and Low (< 0.1 µg/m^3^) inhalable CNT/F exposures in the industrial setting. Peptide biomarker model building and refinement employed linear regression and partial least squared discriminant analyses. Top-ranked peptides were then sequence identified and evaluated for pathological-relevance.

**Results:**

In total, 41 peptides were found to be highly discriminatory after model building with a strong linear correlation to personal CNT/F exposure. The top-five peptide model offered ideal prediction with high accuracy (Q^2^ = 0.99916). Unsupervised validation affirmed 43.5% of the serum peptidomic variance was attributable to CNT/F exposure. Peptide sequence identification reveals a predominant association with vascular pathology. ARHGAP21, ADAM15 and PLPP3 peptides suggest heightened cardiovasculature permeability and F13A1, FBN1 and VWDE peptides infer a pro-thrombotic state among High CNT/F workers.

**Conclusions:**

The serum peptidome affords a diagnostic window into sub-symptomatic pathology among CNT/F exposed workers for longitudinal monitoring of systemic health risks.

**Graphical abstract:**

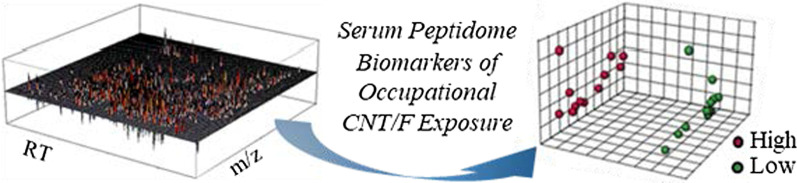

**Supplementary Information:**

The online version contains supplementary material available at 10.1186/s12989-021-00431-0.

## Introduction

Use of carbon nanotubes and nanofibers (CNT/F) continues to grow across multiple industries, from aerospace, automotive, and electronics to healthcare and the life sciences [1]. Their unique physicochemical properties make CNT/F attractive for a variety of manufacturing needs; however, the recent growth in their use presents unknown occupational health hazards. Mounting evidence from animal studies link pulmonary nanoparticle exposure to a diversity of pathophysiological consequences from inflammation, lung fibrosis and cancer, to cardiovascular dysfunction and neurological deficits [2–7]. Thus, there is concern over long-term implications for CNT/F workers, substantiating a need for novel biomarkers to detect and assess health outcomes longitudinally.

Determining CNT/F toxicity is a non-trivial task given varied physical and chemical properties across a diversity of nanoparticles, which influences lung penetration, reactivity, and overall effects in the body [8]. Early epidemiological assessments have focused on acute health ramifications of mixed CNT/F exposures within the industrial setting [9–15]. Not surprisingly, effects were most evident within the lung; yet, Schubauer-Berigan et al. found resting heart rate increased with greater CNT/F exposure, a clinical indication associated with a longer-term increase in mortality [15]. The CNT/F toxicological profile is also influenced by workplace determinants such as environmental conditions, worksite mitigation, and protective equipment measures. Such factors were moderately predictive of CNT/F exposure [16], though cannot inform on the in vivo biological response. In this regard, biomarkers are sought to sensitively detect and quantify pathological effects. Classical cytokine and other protein markers were first assessed as indicators of inflammatory and oxidative processes; however, protein levels were either undetectable [14] or insensitive to the array of potential CNT/F effects suggested in model systems [17]. Thus, the need remains for discriminatory biomarkers that inform broadly on pathobiological processes and offer improved sensitivity to detect sub-symptomatic findings that may prime chronic disease.

To this end, we recently reported the discovery of a complex peptidomic response translated between the lung and general circulation after modeled multi-walled carbon nanotube (MWCNT) exposure in mice. Proteolytic processes within the lung resulted in peptide fragments entering the circulation where they acted as cell surface receptor ligands [18]. The isolated serum peptidome from MWCNT-exposed mice induced endothelial inflammation and vascular dysfunction, affirming its role in broadly promoting systemic health outcomes. Given the serum peptidome’s robust quantitative response to modeled CNT exposure and its pathobiological relevance, studies here assessed the serum peptidome’s diagnostic potential in a cross-industry group of CNT/F exposed workers.

Studies here were facilitated by an industrywide, cross-sectional, epidemiological program conducted by the National Institute for Occupational Safety and Health (NIOSH), which collected personal breathing zone measures, health and demographic information, and matched serum specimens from 102 CNT/F workers across twelve primary and secondary manufacturing sites in the U.S. [19] Previous reports from this program demonstrated that protein markers of fibrosis and oxidative stress in worker sputum indicated a pulmonary insult; however, blood biomarkers of systemic pathologies were inconsistently correlated with exposure metrics [14]. Dose-dependent inflammatory effects were later affirmed in serum using a more sensitive ex vivo assay from Myriad RBM called TruCulture, which used a secondary immunological challenge in cell culture to more ably detect the pro-inflammatory potential of serum [17]. Though the nature of the assay prevented assessing a broader array of pathologies, the results nicely illustrated the potential of and continued need for highly sensitive pathogenic measures relevant to chronic disease. The present study evaluated the serum peptidome – the complement of peptide fragments augmented within the blood – as a novel biomarker source for both the indirect measurement of inhaled CNT/F and early detection of health effects. As observed in animal modeled MWCNT exposure, we posited that sub-clinical pathobiology within the lung would quantifiably alter the serum peptidome and allow effective discrimination with an in vivo assessment of worker CNT/F exposure. This study used a stratified sampling design of matched High and Low (below the limit of quantification) CNT/F exposed worker cohorts for efficient biomarker discrimination [14, 15]. Moreover, as peptide fragments were previously shown to be consequent pathological remodeling of tissue in the lung and vasculature, we anticipated that their detection would be functionally relevant to early health outcomes in exposed workers. Thus, goals of this study were to determine whether a robust serum peptidomic response could be detected among CNT/F industry workers, whether those peptides were relevant to pathobiological outcomes suggested in modeled research, and to assess the diagnostic potential of the serum peptidome as an in vivo indicator of greater occupational CNT/F exposure. Positive findings here would be advantageous among occupational fields in primary and secondary CNT/F manufacturing and likely other areas with exposure to high-aspect nanomaterials.

## Results

### CNT/F occupational exposure induced a significant and diverse peptidomic response in the blood

In this study, the serum peptidome was assessed for 24 workers at CNT/F facilities who were not active smokers and reported no active respiratory disease (mass spectrometry dataset available via the MassIVE repository, MSV000087305, of the ProteomeXchange Consortium, PXD025646). Subjects divided equally into two exposure groups based on their personal breathing zone levels of inhalable elemental carbon (EC): High exposure subjects had > 0.5 µg/m^3^ inhalable EC; Low exposure subjects had measures below the 0.13 µg/m^3^ limit of quantification for inhalable EC (three standard deviations from the limit of detection) [19]. In this regard, subjects within the Low exposure group were indistinguishable from non-exposure. Since we could not rule out cross-contamination into non-production areas where these workers did administrative/office jobs, we conservatively called this cohort a low exposure rather than non-exposed group. All three CNT/F exposure metrics were significantly greater for the High relative to the Low exposure groups (Table 1): inhalable EC, Kruskal Wallis H = 17.28, p < 0.001; respirable EC, H = 17.28, p < 0.001; CNT/F structure counts, H = 6.16, p = 0.013. Additionally, the average respirable EC for the High group exceeded the 1.0 µg/m^3^ NIOSH recommended exposure limit (REL) [14]. Subjects were all exposed to high-aspect nanomaterials: 83% MWCNT, 16% carbon fiber, and 8% single-walled CNT materials, with the later cohorts being too small to assess differences. A detailed physiochemical characterization of these materials has been separately published [8]. Demographics, health history, and reported solvent exposure were all closely matched between groups (Table 1). Lastly, the pro-inflammatory potential of each subject’s blood was assessed using the serum cumulative inflammatory potential (SCIP) assay, measuring pro-inflammatory gene induction within human vascular endothelial cells after incubating with a worker’s serum. SCIP results for endothelial IL6, CCL2, TNFalpha, VCAM or ICAM (Table 1) showed no significant difference in the inflammatory potential of blood from High and Low exposure groups.

**Table 1:** CNT/F worker demographics, exposure, and SCIP metrics for low and high exposure groups

Worker demographics and exposure metric data	Exposure groups	
	High	Low
*Demographics*		
Age [binned 1–5]^a^, ${\bar{\text{x}}}$¯ (SE)	3.0 (0.4)	3.0 (0.3)
Sex, # Females, n (%)	3 (25)	2 (17)
Education [binned 1–4]^b^, ${\bar{\text{x}}}$¯ (SE)	2.8 (0.4)	3.2 (0.3)
Former Smokers, n (%)	3 (25)	2 (17)
Allergies, n (%)	5 (42)	5 (42)
Hypertension, n (%)	3 (25)	2 (17)
Current solvent Exp., n (%)	7 (58)	6 (50)
Past solvent Exp., n (%)	6 (50)	5 (42)
*CNT/F exp*		
Inhalable EC [µg/m^3^], ${\bar{\text{x}}}$¯ (SE)	8.706 (5.402)	0.055 (0.014)
Respirable EC [µg/m^3^], ${\bar{\text{x}}}$¯ (SE)	1.564 (0.964)	0.017 (0.006)
CNT/F structure Count [s/cm^3^], ${\bar{\text{x}}}$¯ (SE)	0.442 (0.310)	0.005 (0.002)
CNT/F duration [binned 1–5]^c^, ${\bar{\text{x}}}$¯ (Se)	3.3 (0.3)	2.4 (0.4)
*SCIP assay*		
IL6 log fold-change, ${\bar{\text{x}}}$¯ (SE)	0.958 (0.094)	0.980 (0.069)
CCL2 log fold-change, ${\bar{\text{x}}}$¯ (SE)	0.830 (0.077)	1.001 (0.099)
TNFalpha log fold-change, ${\bar{\text{x}}}$¯ (SE)	1.231 (0.187)	0.950 (0.102)
VCAM log fold-change, ${\bar{\text{x}}}$¯ (SE)	1.159 (0.178)	1.154 (0.141)
ICAM log fold-change, ${\bar{\text{x}}}$¯ (SE)	0.907 (0.128)	1.228 (0.247)

${\bar{\text{x}}}$¯, mean; SE, standard error; n, sample size; EC, elemental carbon; CNT/F, carbon nanotubes and nanofibers; SCIP, serum cumulative inflammatory potential

^a^Age binning (years): 1 (< 25), 2 (25- < 35), 3 (35- < 45), 4 (45- < 55), 5 (55- < 65)

^b^Education binning: 1 (high/trade school), 2 (some college), 3 (college grad), 4 (postgraduate)

^c^CNT/F exposure duration binning (years): 1 (0), 2 (0.01- < 1), 3 (1- < 5), 4 (5- < 10), 5 (≥ 10)

**Fig. 1:**
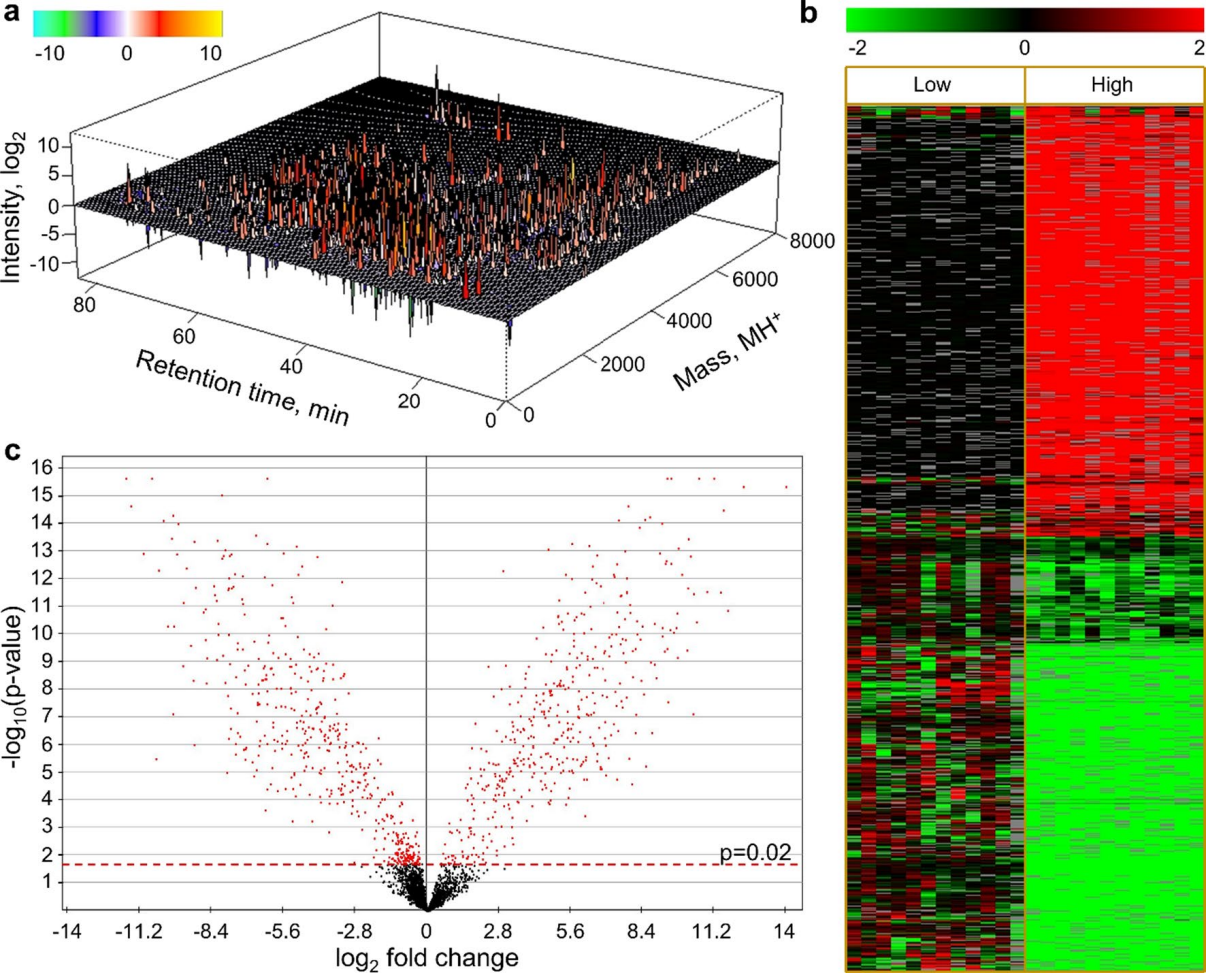
The serum peptidome discriminated workers with acute CNT/F exposure. The serum peptidome was quantified across manufacturing workers grouped by Low (< 0.1 µg/m^3^ inhalable EC fraction) and High (> 0.5 µg/m^3^ inhalable EC fraction) CNT/F exposure across two workdays. **a** Relative peptide ion intensity is perspective-plotted by reduced mass and chromatographic retention time measures, illustrating significant differences between High and Low exposure groups. **b** Relative quantity across individual workers is shown by heatmap for the 934 statistically responsive serum peptides detected in ≥ 75% of workers. Data are plotted as log(2) fold change from the Low-group mean. **c** The serum peptidome response as illustrated by a volcano plot of fold-change from the Low-group mean plotted against p-values, with the significance threshold adjusted to a 5% FDR

Quantities of each serum peptide were tabulated across all workers. In total, 11,546 peptide features were reproducibly quantified in ≥ 50% of workers, with 2,726 achieving significance between High and Low groups after correcting for multiple measures. Detected features spanned broadly across the available separation space (Fig. 1a), demonstrating that these results were not artefactual from sporadic interference or insufficient separation. Given the serum peptidome’s diverse response and the goal to identify group-discriminant peptides, the dataset was filtered further to retain the 3,532 peptides reproducibly detected in ≥ 75% of workers, which achieved an estimated 77% power to discriminate a two-fold group-difference (see Additional file 1). Of those peptides, 934 were statistically significant between High and Low groups (Fig. 1b,c), providing a reliable input dataset for model building. Overall, results here substantiate a significant and diverse serum peptidomic response conserved among workers with an average exposure exceeding the NIOSH 1.0 µg/m^3^ REL for CNT/F.

### CNT/F exposure-responsive serum peptidome model building

**Fig. 2:**
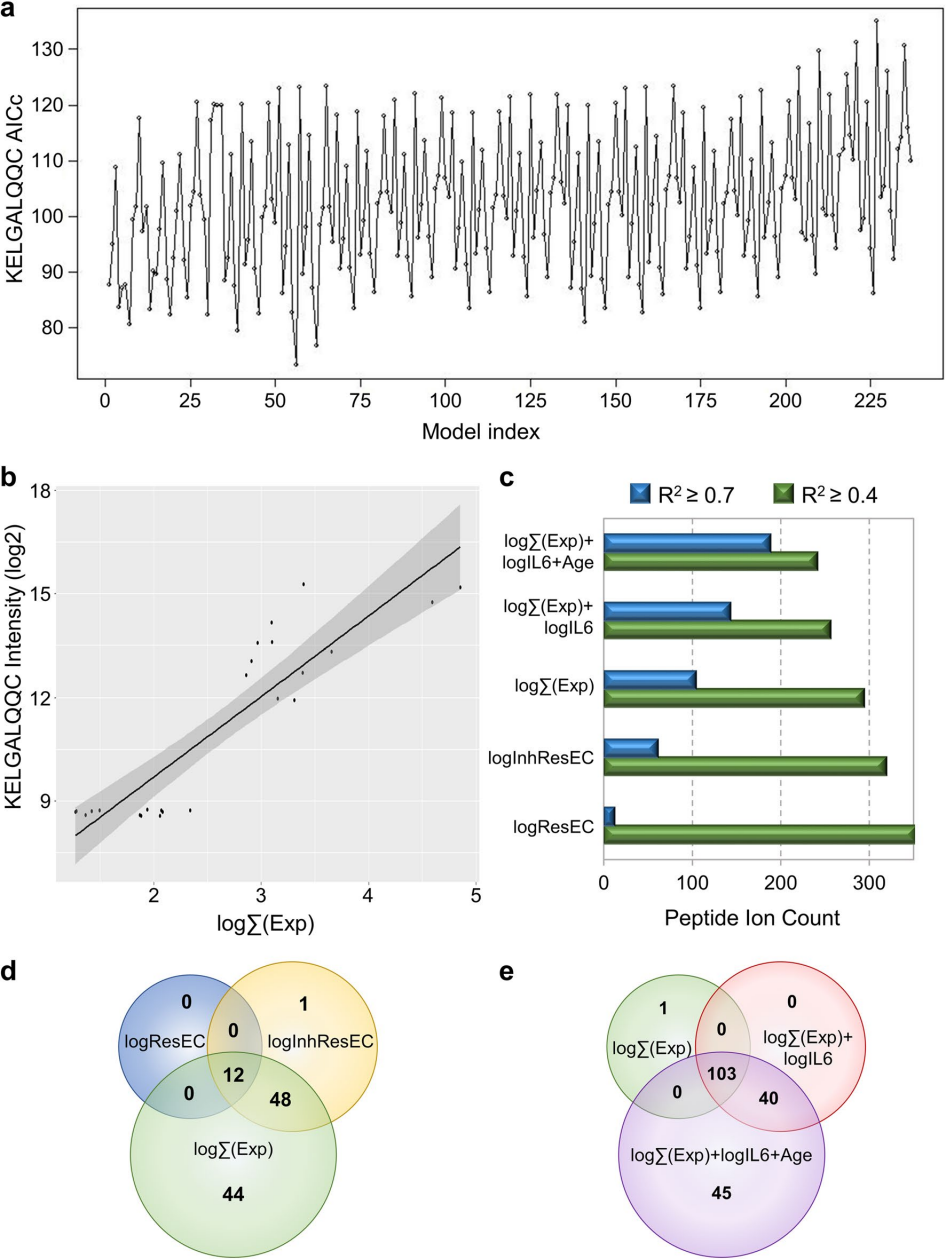
Serum peptidome biomarker model building with linear correlated features of worker CNT/F exposure. The 934 significantly responsive serum peptides were assessed by multivariate linear regression against metrics of CNT/F breathing zone levels, worker demographics and histories, and assay measures of serum inflammatory potential. **a** Out-of-sample predictive error was assessed by Akaike information criterion (AICc) scores for multivariate worker metric combinations. **b** Performance was best when taking the linear combination of three personal breathing zone CNT/F measures, log∑(Exp), with linearity exemplified for peptide KELGALQQC. **c** Moderate correlation (Shapiro R^2^) was observed across 39% of the responsive peptides for respirable EC measures (logResEC), with performance improved when adding inhalable EC (logInhResEC) and CNT/F structure counts (log∑(Exp)). Additional serum peptidome variability was explained by covariates for serum induction of IL6 and worker age. **d-e** Venn diagrams illustrating the overlap of strongly correlated (R^2^ > 0.7) serum peptides between multivariate worker metrics

The 934-peptide subset was assessed for correlation with the three CNT/F exposure metrics alone, in combination, and together with covariates of exposure duration, age, and SCIP assay measures (Table 1). Goodness-of-fit for each peptide was considered using 237 Akaike information criterion (AIC) scores for each variable combination (exemplified in Fig. 2a). Low AIC scores indicated minimal information loss (out-of-sample prediction error); thus, variable combinations having the greatest number of low-scoring AIC peptides provided the best fit to the peptidomics data. Relative to random scoring, 5 variable combinations exhibited a significant peptidome association: respirable EC (logResEC) alone matching 214 peptides (Z = 3.84, q = 0.0029); the linear combination of logResEC and inhalable EC (logInhResEC) matching 357 peptides (Z = 7.13, q < 0.0001); the linear combination of logInhResEC and CNT/F structure counts (log∑Exp) matching 492 peptides (Z = 11.22, q < 0.0001); the combination of log∑Exp with logIL6 SCIP assay data (log∑Exp + logIL6) matching 292 peptides (Z = 5.55, q < 0.0001); the combination of log∑Exp + logIL6 and subject age (log∑Exp + logIL6 + Age) matching 268 peptides (Z = 5.01, q < 0.0001). Other covariates such as sex and solvent exposure did not change the parameter estimates, even with the groups matched across these dichotomous variables. These results indicate that more of the CNT/F peptidomic response was explained when combining all three personal breathing zone exposure metrics into a composite log∑Exp value, which may better reflect overall lung burden. The addition of other covariates to log∑Exp such as the logIL6 measure of blood pro-inflammatory potential or subject age did not strengthen the overall fit.

Next, we assessed the linear correlation between the 5-best AICc scoring variable combinations mentioned above and the 934-peptide subset (exemplified in Fig. 2b). All combinations exhibited moderate correlation (R^2^ ≥ 0.4) across a sizable proportion of the serum peptidome (Fig. 2c). However, the number of peptides with strong correlation (R^2^ ≥ 0.7) was substantially increased with the linear combination of CNT/F exposure metrics – from just 12 peptides for logResEC alone on up to 104 for log∑Exp combination of all three CNT/F metrics (Fig. 2d). The addition of covariates for pro-inflammatory potential (logIL6) and subject age strengthened the linear correlation further, adding an additional 40 and 45 peptides, respectively (Fig. 2e). However, since a large portion of the co-linear peptides were in common across these three variable combinations (Fig. 2e), we decided to focus on those 103 common peptides, avoiding the concern that orthogonal covariates can artificially inflate R^2^ values.

### CNT/F exposure-responsive serum peptidome model refinement and evaluation

**Fig. 3:**
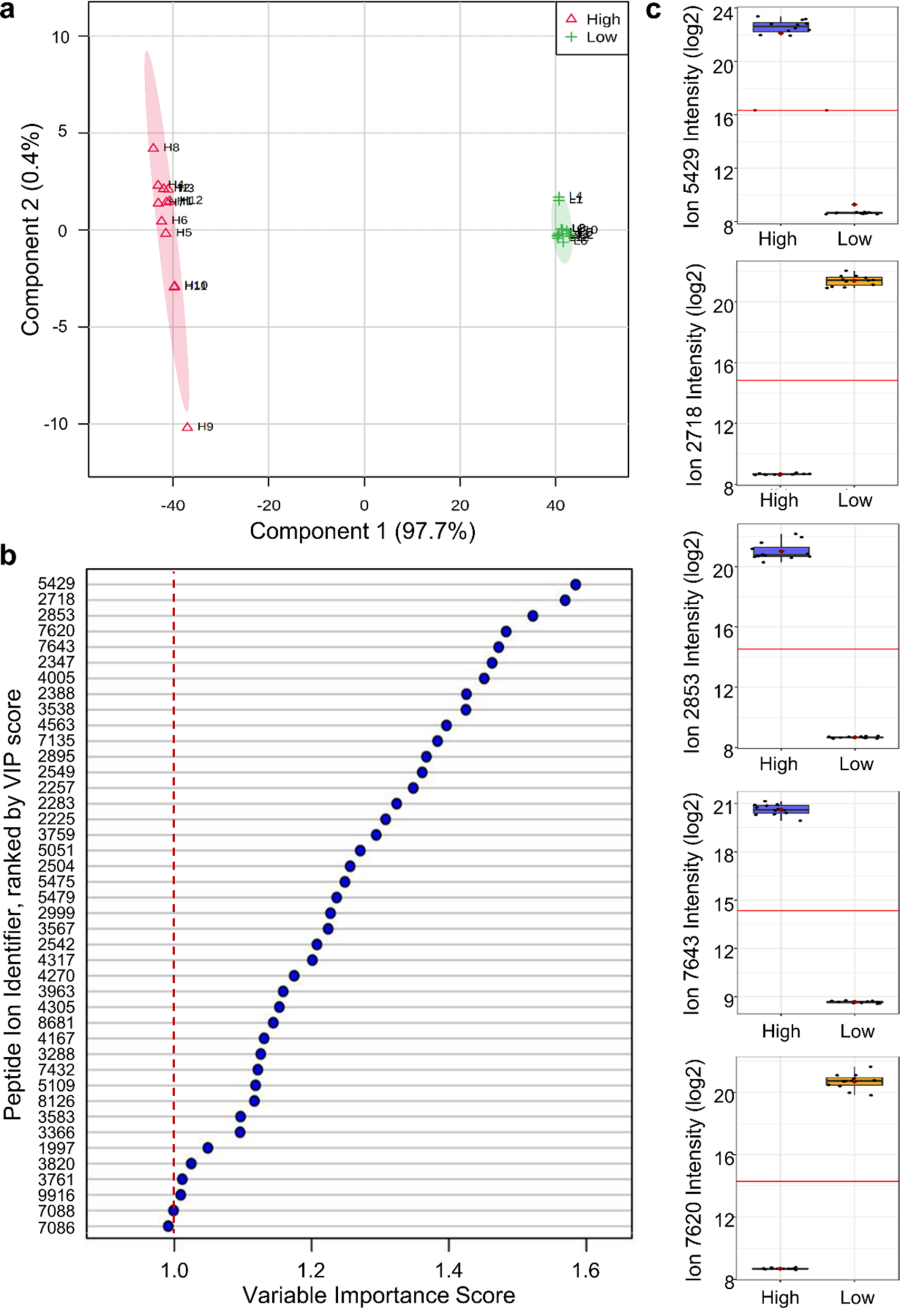
Discriminant peptide ranking for biomarker model refinement. PLS-DA was employed to select the most predictive peptide measures that **a** resolved High CNT/F and Low CNT/F exposure groups. **b** The rank of each peptide measure’s contribution to the PLS-DA model was provided by the weighted sum to the squared correlation as a VIP score (peptide measures shown by their unique index identifier). Using an accepted 1.0 VIP cutoff, 41 peptide measures were found most discriminatory between groups. **c** Box plot of the top five scoring peptide measures between High and Low CNT/F exposure groups

Model refinement was conducted using supervised partial least square discriminant analysis (PLS-DA) to rank peptides that provided maximal discrimination between High and Low exposure groups (Fig. 3a). Using a variable importance in projection (VIP) score cutoff of 1.0 [20], 41 of the 103 peptides were prioritized (Fig. 3b). The top five peptides alone exhibited between a 3 and 4 order difference in quantified peptide ion intensity between High and Low exposure groups (Fig. 3c), denoting sensitive detection over a wide dynamic range.

**Fig. 4:**
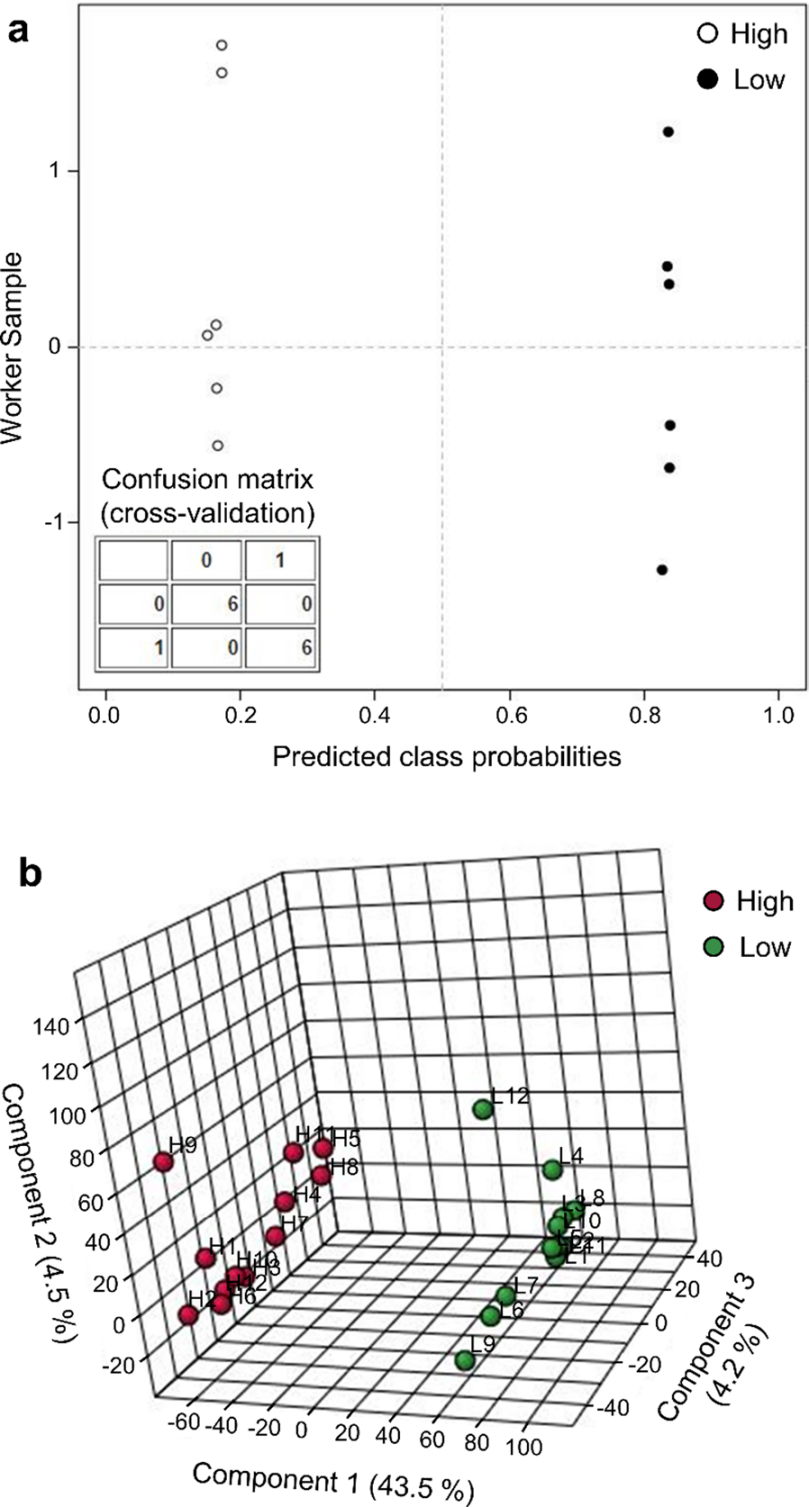
Validation of the serum peptidome biomarker model for classifying CNT/F worker exposure. **a** The performance of the top-five peptide model was assessed by cross-validation where the average class probabilities for validation subjects were plotted. Ideal classification was demonstrated as illustrated within the confusion matrix table. **b** To rule out model over-fitting, the full complement of 3,532 reproducibly detected peptides (without any supervised selection) was assessed by unsupervised principal component analysis where 43.5% of the serum peptidome variance could be explained by worker CNT/F exposure level

Next, a cross-validation of the top-five peptide model was performed using a Monte Carlo leave-n-out procedure to assess generalized predictive capability. Predictive accuracy remained ideal at 1.0, with a Q^2^ statistic of 0.99916, demonstrating retained predictive power across permutations. Next, cross-validation assessment was performed on a validation subset of 6 subjects per group with data entirely separate from those use for top-five model building. Ideal discrimination was observed with no misclassification after 50 random sub-samplings between workers with > 0.5 µg/m^3^ inhalable CNT/F exposure from those with < 0.1 µg/m^3^ (Fig. 4a).

Unsupervised principal component analysis (PCA) was then performed to further affirm the discriminatory power of the serum peptidome and rule out overfitting of the supervised procedures used to build the model. When assessed across the full complement of 3,532 highly reproduced peptides, without any supervised testing applied (Fig. 4b), the results clearly discriminated workers with High from Low CNT/F exposure across principal component 1, with 43.5% of the peptidome variability explained. Results demonstrate that the serum peptidome exhibited latent differences based on the magnitude of exposure, with more modest principal components offering within-group discrimination that appeared irrespective to CNT/F exposure. Taken together, these data indicate that the circulating peptidome possessed strong latent discriminatory capacity and could be diagnostic of occupational CNT/F exposure.

### Discriminatory peptides suggest sub-symptomatic CNT/F-mediated pathology

**Fig. 5:**
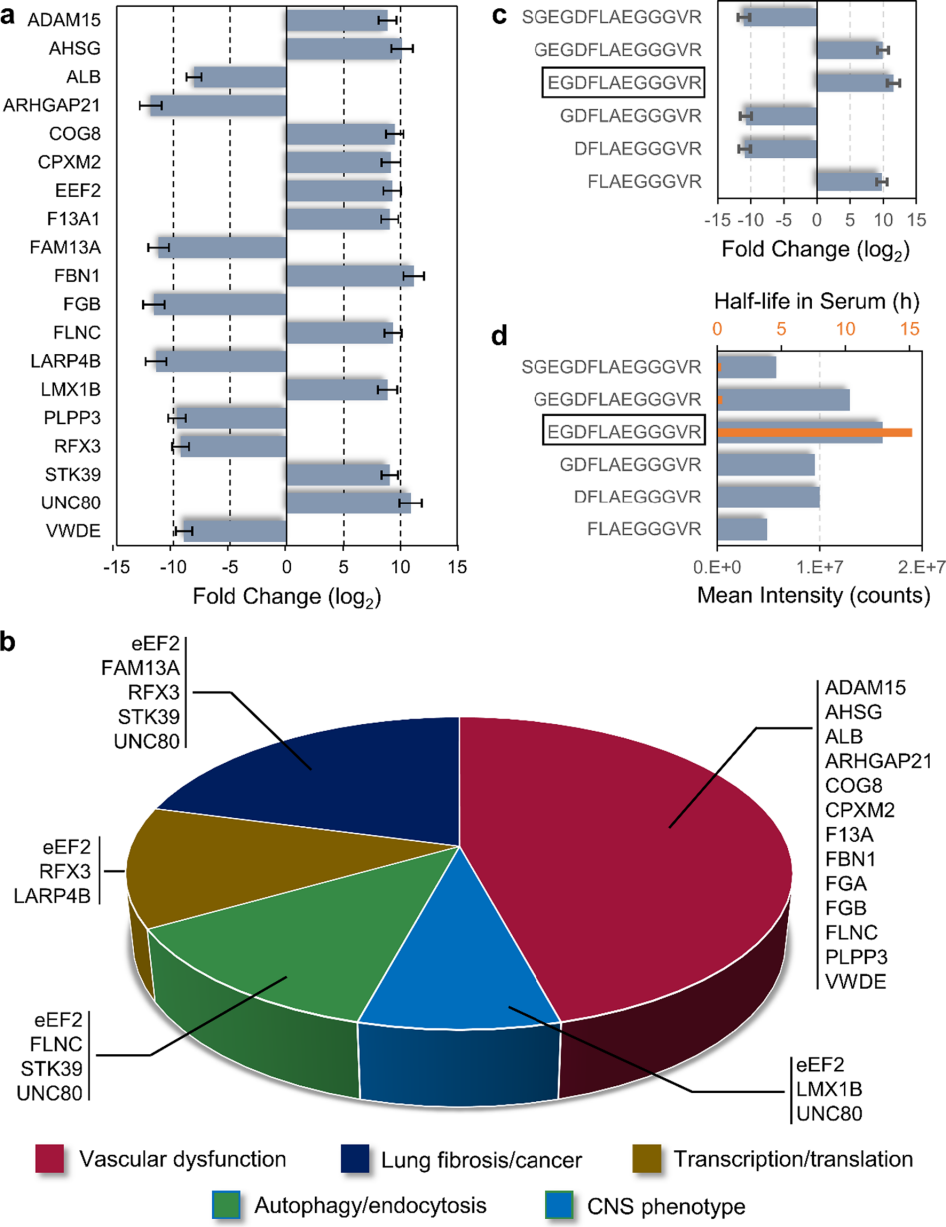
Identification and functional relevance of the 41 top-ranked discriminant peptides. **a** Fold-change response of the identified discriminant peptides grouped by their originating protein symbol (excluding FGA). Mean ± S.E., q-value < 0.05, normalized to the Low group mean. **b** Functional classification of the 20 parent proteins for the identified discriminant peptides. **c** Fold-change response for six identified fibrinopeptide A fragments per normal exoproteolytic serum metabolism, with the most stable (15.2 h half-life) shown boxed. Mean ± S.E., q-value < 0.05, normalized to the Low group mean. **d** Plot of the mean peptide ion intensity across all study subjects (gray) and the half-life in hours (orange, values from Yi et al*.* [28]) for the six identified fibrinopeptide A fragments

As introduced earlier, nanoparticle exposure in animal models produced systemic pathology, particularly for the cardiovascular system [21] along with potential neurological consequences [22, 23]. However, epidemiological evidence for such health outcomes has been inconclusive. Thus, the functional relevance of the top 41 CNT/F exposure discriminating peptides was of particular interest as to whether they reflected ongoing sub-symptomatic pathobiology. Utilizing the generated tandem mass spectra, 27 of the 41 discriminant peptides were identified (see Additional file 2) with sequences derived from 20 parent proteins (Fig. 5). Functional annotation revealed the largest cohort of 12 factors were associated with vascular dysfunction (Fig. 5b). For example, ADAM15 (a disintegrin and metalloproteinase domain-containing protein 15) is known to enhance endothelial hyperpermeability with a corresponding 8.88-fold peptide increased [24]. Additionally, we observed a significant 9.70-fold decrease in a peptide from phospholipid phosphatase 3 (PLPP3), a protein otherwise known to be protective against endothelial dysfunction [25]. Similarly, a peptide from Rho GTPase-activating protein 21 (ARHGAP21) was reduced 12.02-fold, which would implicate disinhibition of Rho kinase and increased vascular permeability [26]. Also among vascular-related proteins was fibrinogen alpha chain (FGA) with seven of the 27 identified peptides. Six of the FGA peptides were exoproteolytic fragments of fibrinopeptide A (FpA), each with starting positions between FGA residues 19 and 27, but all terminating at the thrombin-cleaved arginine residue 35 (Fig. 5c) [27]. N-terminal FpA truncation occurs naturally with degradative clearance in the blood [28]. However, the fragment EGDFLAEGGGVR exhibits the longest half-life (Fig. 5d), 52-fold that of full-length FpA. Thus, this fragment has been found the most stable and best FpA biomarker when resolved by mass spectrometry. Moreover, we found that it had the overall highest intensity across all subjects (Fig. 5d) and was significantly increased 11.57-fold between High vs Low CNT/F exposure groups. Importantly, an FGA peptide not associated with FpA (SSSYSKQFTSSTSYNRGDSTFESKSY, residues 575 and 601) was also increased (8.50-fold) between Low and High CNT/F exposure cohorts. This peptide is generated via carboxypeptidase B2 cleavage, which is itself activated by thrombin to downregulate fibrinolysis and exacerbate thrombotic conditions [29]. Together, these results suggest greater vascular permeation and a pro-clotting phenotype among High CNT/F exposed workers.

The next largest group of five proteins associated with lung pathologies. For example, an 11.32-fold decrease in a peptide from FAM13A (family with sequence similarity 13 member A) was consistent with lung fibrosis-associated FAM13A down regulation [30]. A peptide to RFX3 (transcription factor RFX3) implied a decrease in the protein, which is known to stunt cilia formation, motility and impair mucosal clearance [31]. On the other hand, increased expression of eEf2 (eukaryotic translation elongation factor 2), UNC80 (unc-80 homolog), and STK39 (STE20/SPS1-related proline-alanine-rich protein kinase) have been associated with non-small cell lung cancer [32, 33] and tumorgenesis [34, 35], with corresponding peptides found here increased by 9.29-fold, 10.92-fold and 9.08-fold, respectively. Thus, as seen with vascular-related peptides, these measures may offer sensitive indication of sub-symptomatic pathology consistent with CNT/F exposure in animal models.

## Discussion

This study assessed whether circulating peptides (the serum peptidome) provided evidence of sub-symptomatic systemic pathobiology following worker-exposure to CNT/F nanoparticulate, and whether that response exhibited latent diagnostic value. We recently published that CNT/F exposure in mice augmented the serum peptidome to include bioactive proteolytic fragments that induced vascular dysfunction and inflammation [18]. Results here are the first to substantiate an equally diverse and intense shift within the serum peptidome among a group of occupationally exposed workers where the average respirable CNT/F EC fraction (1.564 µg/m^3^) approximated the current NIOSH REL of 1.0 µg/m^3^. The induced serum peptidomic change was highly consistent within the High CNT/F exposure cohort, with 934 significant peptides detected in ≥ 75% of the twelve workers, and 2,726 in ≥ 50%. Top discriminatory peptides responded on average by 3 to 4 orders in magnitude between High and Low exposure cohorts, with estimated serum concentrations for the 27 identified top discriminatory peptides (Additional file 2) ranged from 0.13 nM (effectively the limit of quantification) to as much as 600 nM. This level of sensitivity between groups along with the high level of consistency across workers supports the rigorous diagnostic potential of the serum peptidome in response to CNT/F and, expectedly, other inhalation exposures to high-aspect nanoparticulates. Past companion studies were hampered by the general low level of exposure across the 102 workers [14, 17]. The stratified sampling design used here provided greater efficiency with which 41 highly discriminatory peptides were resolved. These 41 peptides were all strongly correlated (R^2^ > 0.7) with the composite measure of personal breathing zone CNT/F and were highly accurate at subject classification.

Mixed linear models were used to consider each of the three CNT/F exposure metrics alone, in combination, and with other demographic and worker history covariates. Additionally considered was the inflammatory potential of a worker’s serum, since prior results in mice suggested that CNT/F exposure can drive systemic endothelial inflammation [36]. Results showed that goodness-of-fit was maximized through the linear combination of respirable and inhalable elemental carbon measures together with the count of CNT/F structures per air volume (log∑Exp). This finding was consistent with prior companion studies that found the inhalable fraction and structure count measures significantly associated with biomarker and health outcome results, often independently from measures of respirable elemental carbon, the current basis of the NIOSH regulatory exposure limit [14, 15, 17]. However, ours was the first study to model the linear combination of all three exposure metrics, which provided better correlation with the serum peptidomic response and may help guide revised regulatory limits after further confirmatory studies on the dose–response relationship. In total, 298 peptides provided moderate correlation (R^2^ > 0.4) with log∑Exp, of which 104 were strongly (R^2^ > 0.7) correlated. Additional variance within the serum peptidome could be explained with the added covariates of subject age and the ex vivo measure of serum-induced endothelial IL6, while appearing unconfounded by other covariates of sex and past solvent exposure. Interestingly, Schubauer-Berigan et al*.* found that age was a significant demographic covariate relevant to their assessment of CNT/F worker serum inflammatory potential [17]. Together, these findings justify follow-up studies on the serum peptidome with a larger, more diverse worker population as needed to investigate covariates with greater power and to assess other susceptibility factors that could not be studied here; e.g., nanoparticulate properties, mixture interactions to include solvents, as well as worker genetic background, life-stage, life-style factors, and health status [37]. Moreover, modes of exposure with different job tasks and the use of personal protective equipment greatly influence the internal CNT/F burden, which we’ve shown previously to vary greatly across facilities [16, 19]. Yet, it should also be noted that conducting a larger epidemiological study on CNT/F occupational exposure within the US would be challenged by the small (though growing) industrial footprint and generally effective environmental controls limiting the range of exposures.

To avoid overinflating results, we conservatively limited further development to those 103 peptide factors that retained strong linearity with log∑Exp alone or in combination with logIL6 and age factors. All told, worker log∑Exp values explained 97.7% of the serum peptidomic variance among the 103 peptides, demonstrating their high selectivity, with exposure metrics background corrected for non-CNT/F ambient particulate [19]. Cross-validation demonstrated that model performance was maintained with 100% accuracy using the top five ranked peptides, having a Q^2^ statistic of 0.99916. Similarly, when employing separate worker subsets for model building and validation, the top five peptide model provided ideal classification into High and Low exposure cohorts per predictive class probabilities. We independently applied unsupervised principal component analysis to rule out overfitting of high-dimensional data with PLS-DA [38]. Indeed, 43.5% of the variance across all 3,532 reproducible peptide measures, without a priori supervised statistical selection, was explained by a single PCA component, which provided unambiguous stratification of the 24 workers into High and Low exposure cohorts. These data demonstrate the quantitative and predictive capacity of a multivariate peptidomic biomarker assay to inform on occupational CNT/F exposure.

Peptide sequence identification provided insight into pathobiological relevance. Of the 41 VIP ranked peptides, 65% (13 of 20) of the corresponding proteins associated with vascular dysfunction and thrombotic responses, which was the most enriched functional association and was complementary to results from the prior TruCulture secondary immuno-challenge assay study [17]. Two of these proteins, fibrinogen and von Willebrand factor (vWF or VWDE), were assessed previously among CNT/F workers (Table 2). Peptide results here suggested a decrease in vWF that was consistent with Beard et al*.*’s observed decrease in the serum protein across the full cross-sectional parent study [14], though Kuijpers et al*.* had found no overall trend in this protein across a smaller cohort of workers [39]. Notably, a comparable vWF decrease was observed in our peptidomic study of modeled CNT/F exposure in mice (Table 2) [18]. Likewise, fetuin-A (AHSG), fibrillin-1 (FBN1) and fibrinogen measures were directionally consistent between the present human study and the prior mouse study. Fetuin-A increased with CNT/F exposure, a response positively associated with a wide range of cardiovascular outcomes including atherosclerosis, myocardial infarction, and arterial thickening [40–42]. Fibrillin-1 was also increased with CNT/F exposure, which has been reported to enhance arterial and myocardial thickening and hypertension [43].

**Table 2:** Cross-study comparison of cardiovascular-related blood biomarker responses to CNT/F exposure

Serum protein biomarker (or peptide parent)	Liou et al. (2012)	Liao et al. (2014)	Kuijpers et al. (2018)	Beard et al. (2018)	Mostovenko et al. (2019)	Current study
	Cross-sectional	Longitudinal	Cross-sectional	Cross-sectional	Mouse Model	Case–control
ADAM15						Up
Fetuin-A					Up	Up
Albumin					Up & Down	Down
ARHGAP21						Down
COG8						Up
CPXM2						Up
F13A						Up
Fibrillin-1					Up	Up
Fibrinogen-A	Up	Unchanged	Unchanged	Up & Down	Down	Down
Fibrinopeptide-A					Up & Down	Up & Down
FLNC						Up
ICAM-1	Up	Unchanged	Up	Up		
PLPP3						Down
t-PA			Unchanged	Down		
VCAM-1	Unchanged	Up	Unchanged	Up		
vWF (VWDE)			Unchanged	Down	Down	Down

Fibrinogen (FGA) results were more complex to interpret and to compare with prior studies. Seven FGA peptides were identified with all but one related to fibrinopeptide A (FpA). Results from prior CNT/F worker studies, however, did not differentiate between whole protein and fibrinopeptide, an important consideration given that FpA is a thrombin activity marker distinct from whole FGA levels [28]. Found here was an FpA peptide fragmentation series consistent with exoproteolytic metabolic clearance in the blood. Yi et al*.* determined that EGDFLAEGGGVR was the most stable fragment due to its charged starting residue (glutamic acid), exhibiting the longest half-life (15.2 h) in the blood, and making it the preferred FpA biomarker (when resolved by mass spectrometry) for assessing thrombin activity. Results here showed a significant increase in this FpA fragment among High CNT/F workers, indicating elevated thrombin activity. Moreover, we detected a c-terminal fragment of FGA that demarked elevated carboxypeptidase B2 activity within the blood of High CNT/F workers, a serum protease otherwise known as the thrombin-activatable fibrinolysis inhibitor [29]. As the alternative name implies, detection of this proteolytic product indicated clot lysis inhibition. Together, the peptidomic response to CNT/F exposure symbolized a pro-thrombotic state that warrants follow-up affirmation.

Another peptide that was increased among High CNT/F workers hailed from the thrombin cleavage site of coagulation factor XIII (F13A1) and provided further indication of heightened thrombin activity in the blood. Beyond clot formation, thrombin also acts to attenuate Rho GTPase activity and promote MLC2-mediated vascular hyperpermeability [44–46]. Peptidomics data here provides evidence for this process occurring among High CNT/F workers. An observed peptide to Rho GTPase activating protein 21 (ARHGAP21) suggests Rho kinase disinhibition that permits MLC2-driven vascular permeability [26] as seen after PM2.5 inhalation [47]. Additionally, a significant decrease in the peptide from phospholipid phosphatase 3 (PLPP3) implied a loss of this protein, which enhances endothelial dysfunction and permeability [25]. Furthermore, High-group CNT/F workers exhibited an increase in a peptide to ADAM15, a matrix protease known to induce vascular hyperpermeability [24]. Interestingly, the ADAM15 peptide was tyrosine sulfated, a modification occurring in the Golgi that directs protein secretion [48]. ADAM15 is further regulated in the Golgi as a glycosylation substrate of COG8 (conserved oligomeric Golgi complex subunit 8) [49], which itself had an increased peptide among High-group CNT/F workers. Thus, in aggregate, the CNT/F-exposure discriminating peptides predominately reflected a pro-thrombotic state with thrombin-related endothelial hyperpermeability, as suggested with modeled exposures to fine particulate matter (PM2.5) [50] and CNT/F [51].

A smaller cohort of peptides may relate to lung pathology and potential carcinogenesis. Decreased FAM13A peptide levels were consistent with a decrease in the protein during lung fibrosis [30]. At the same time, elevated peptide levels suggest greater STK39 in the blood, a marker of oncogenesis [35]. eEF2 peptide levels increased, with elevated eEF2 protein demarking non-small cell lung cancer [32]. eEF2 along with LAPR4B dynamics have also been tied to disrupted basement membrane integrity in the lung epithelium [52], which would present access to the circulation. Mechanistically, this is tied to phosphorylated eEF2, as indicated by the peptide found here, which suppresses protein synthesis [53] and drives apoptosis [54]. Whereas decreased LARP4B, as suggested by its peptide here, correlates with reduced RNA stability [55] and impaired protein translation [56]. Thus, the peptidomic response also indicates potential pulmonary injury and pro-cancerous pathology consistent with modeled CNT/F exposures.

## Conclusions

In conclusion, this study used well characterized personal breathing zone metrics in a case–control design to identify discriminatory peptides of worker total CNT/F on-the-job exposure. This was the first study to demonstrate the capacity of the circulating peptidome for toxicological diagnostics in humans, finding a diverse and robust response with occupational CNT/F exposure. Identification of the top discriminating peptides reflected not only lung pathology but predominant evidence of vascular abnormality and a pro-thrombotic state that was consistent with pathology induced in animals after modeled CNT/F exposure and a primer for cardiovascular disease and stroke among other chronic maladies. Vascular hyperpermeability findings were consistent with our modeled CNT/F exposure studies, which demonstrated blood–brain barrier disruption accompanied by neurovascular unit and adjoining synaptic circuit abnormalities that were themselves consistent with early pathogenesis in neurodegenerative disease [57, 58]. Yet epidemiological studies to date have found limited evidence for health outcomes among CNT/F workers, as explored recently in a companion study [15]. Schubauer-Berigan et al*.* found associations between CNT/F exposure and increased allergies and resting heart rate. However, findings here offer evidence of sub-symptomatic pathology among the same workers that had exposures near the CNT/F recommended exposure limit. The peptide measures were evident even though the blood of these higher-exposed workers exhibited no measurable inflammatory potential relative to the control group (SCIP assay data), noting that significant SCIP results in animal models were observed at much higher levels of CNT/F exposure, portending the sub-symptomatic sensitivity of the peptidomic measures.

Overall, findings here were correlative, not causative, yet the peptidomic response was robust across a diverse selection of twelve CNT/F exposed workers matched to an equal number of co-workers with undetected exposure. The study was found amply powered for model development; however, the small subject size did limit broader characterization of important covariates like age. The most discriminant model peptides were also highly linear in relation to the log-sum of the three personal breathing zone CNT/F exposure metrics; however, a larger population is again needed for follow-up dose–response characterization. One key consideration here is that with a linear response, these blood-based peptide biomarkers may provide an important (though indirect) tool for measuring internal CNT/F doses, a recognized need in extrapolating suitable dose–response relationships [59]. Setting dose-metric parameters for occupational limits, however, remains highly challenged by the number of covariate factors that may need to be considered (e.g., worker life-stage, genetic background, ongoing pathology) and the diversity of physiochemical properties across engineered nanomaterials in the workplace. Follow-up longitudinal studies are also envisioned to assess the serum peptidomic shift with repeated exposures and its signal longevity when considering adaptive compensatory responses. Results here also indicate a role for sulfation on the serum peptidome, as 5 of 27 identified discriminatory peptides exhibiting this modification. Interestingly, a principal role of sulfation is to direct cellular secretion [47], with Chemokine sulfation induced in response to lung disease [60] and atherosclerosis [61]. Thus, it is reasonable to expect sulfated secreted peptides in response to pulmonary and vascular pathologies induced by CNT/F exposure. In all, the serum peptidome offers a needed diagnostic technology with which to longitudinally follow this at-risk worker population for longer-term pathology, to revise recommended exposure levels, and optimize mitigation measures.

## Methods

### Exposure and sample collection

The studies here were conducted under approval from the NIOSH Institutional Review Board (protocol# 12-DSHEFS-05XP) and with informed consent by all study participants. Subjects were selected from 102 participants in the parent cross-sectional, industrywide epidemiological program of U.S. CNT/F workers as detailed elsewhere [14, 15, 19]. Briefly, workers were recruited from 12 different primary and secondary manufacturing facilities. Workers self-reported current health conditions, sex, age, previous exposure, etc*.* via questionnaire. Personal breathing zone CNT/F was measured across two full work shifts (~ 8 h each) via three different metrics: elemental carbon (EC) mass (µg/m^3^) at the (i) respirable (< 4 µm) and (ii) inhalable (< 100 µm) aerosol particle size fractions as measured using 25-mm cassettes with quartz fiber filters via the NIOSH 5040 analytical method; (iii) inhalable CNT/F structures counted on 25-mm mixed cellulose ester filters by transmission electron microscopy and normalized to the collected air volume as structures per cubic centimeter (s/cm^3^) using a modified 7402 NIOSH analytical method. During the midweek shift for which personal breathing zone exposures were being measured, blood was collected into 5-mL serum separator tubes, which were inverted five times and allowed to clot for 30 min. Tubes were then spun at 1000 g for 10 min at 4 °C to collect serum, aliquoted, and immediately frozen at -20 °C in the field. Samples were then shipped frozen and were maintained at -80 °C until use.

Prior findings with this worker cohort were more often associated with the inhalable EC measure of exposure [14–16]; thus, this metric was used for subject selection as follows. First, subjects (of the 102) were excluded from consideration if they were active smokers (14.7%), had known respiratory disease (asthma, bronchitis, emphysema, or colds, 28.4%), or had recently taken a nonsteroidal anti-inflammatory drug (26.5%). Then all remaining subjects with an inhalable EC measure over 0.5 µg/m^3^ were selected for the “High” exposure group (n = 12). Lastly, an equally-sized set of twelve workers with inhalable EC measures below the 0.13 µg/m^3^ limit of quantification (threefold the detection limit for the EC assay) when using the 25-mm filter at a 3-m^3^ sampling volume were matched across available covariates of age, sex, education, former smoker status, allergies, hypertension and current or past solvent exposure (Table 1) as the “Low” exposure group. We could not rule out CNT/F cross- from production areas to the offices where these workers performed administrative roles; hence, we conservatively called this a Low exposure group even thought it could not be differentiated from non-exposure.

### Serum cumulative inflammatory potential assay

The inflammatory potential of worker serum samples was assessed as previously described [62]. Briefly, naïve human coronary artery endothelial cells (hCAECs; Lonza Allendale, NJ) were platted on 24-well plates and grown to confluence. Cells were then exposed to 5% worker serum for 4 h, then RNA was harvested using an RNeasy Mini Kit (Qiagen, Valencia, CA). Reverse transcription was performed using the High-Capacity cDNA Reverse Transcription Kit (Applied Biosystems, Foster City, CA). The TaqManR Gene Expression protocol was then used for qPCR on select targets (Thermo Scientific, Waltham, MA): *IL6* (Hs00174131_m1), *CCL2* (Hs00234140_m1), *TNF-α* (Hs00174128_m1), *VCAM-1* (Hs01003372_m1), *ICAM-1* (Hs00164232_m1). Gene expression was analyzed with the 2^−ΔΔC^_T_ method and normalized to TATA-box binding protein (Hs00427620_m1).

### Peptidome separation and mass spectrometry

The serum peptidome was resolved with our previously published peptide enrichment procedure [18], modified only to first dilute human serum fivefold with mass spectrometry-grade water ahead of filtration due its greater viscosity relative to mouse serum. In brief, serum was clarified through a 0.22 µm Ultrafree-MC filtration unit (EMDMillipore, Billerica, MA), denatured with 18 mM TCEP and 20% acetonitrile along with the HALT protease and phosphatase inhibitor cocktail (Thermo Scientific, Rockford, IL), and then cysteine alkylated with 30 mM iodoacetamide. Size fractionation was performed on pre-cleaned MicroCon centrifugal filter units (YM-30, EMDMillipore) to collect a < 8 kDa fraction. The retentate was acidified with 0.4% formic acid to disrupt molecular binding and further centrifuged. Samples (4.5 µl with 0.1% formic acid) were then passed through a Symmetry C18 column (Waters, Milford, Massachusetts) for solid phase extraction of lipids, washing of salts, and concentrating of the peptide content. Analytical separation was performed at 55 ºC for 70 min (6% to 35% acetonitrile 0.1% formic-acid) on a NanoAcquity system with a 150 mm × 75 µm HSS T3 capillary column coupled online with a Synapt G2-Si HDMS tandem mass spectrometer with ion mobility enabled (Waters). Data were acquired between 50 and 1950 m z^−1^, with the quadrupole set to filter interferants below 400 m z^−1^, a 25,000 nominal resolution, and the collision energy adjusted to drift time [63]. Spectra peak picking and precursor-to-product binning was performed using PLGS v3.0.3 with ions clustered in EndogeSeq across biological replicates aligned by retention time (± 2 min), drift time (± 4 bins), and charged-reduced ion mass (MH^+^, ± 6 ppm) [18]. The dataset was filtered to retain reproducible ion events observed in 50% or more subjects per group. For quantitative analysis, data were median centered, imputed for non-random left-censored measures at the limit-of-quantification, and log_2_ transformed as described previously [64]. Data were then standardized as fold-difference values from the Low exposure group mean per peptide.

### Statistical analysis, model development and evaluation

Transformed peptidomic data exhibited a normal distribution and were assessed using the student’s t-test, correcting for multiple peptide measures using Benjamini–Hochberg to limit the false discovery rate to 5% in MultiExperimentViewer (v.4.9.0) [65]. Peptides with a significant group difference that were highly-reproducible (present in at least 9 of 12 samples per group) were then assessed with linear regression fitting against subject exposure and clinical metrics [66, 67]. The CNT/F exposure metrics with a lognormal distribution [19] were assessed alone and in combination based on prior observations of biomarker and functional correlation differences in his cross-sectional cohort, and were adjusted for categorical covariates of age, sex and CNT/F exposure duration along with the five continuous gene expression variables from the serum cumulative inflammatory potential assay. Utilizing the R package Rcompanion [68], 237 possible models were fit between the mass spectrometry data and the worker metrics and then evaluated using the compareLM method. Akaike Information Criterion (AICc) scores were calculated per peptide across all models. A count of peptides exhibiting the lowest AICc score *per* model was tabulated, with the count’s significance from random assessed using a one-sample z-test. Models with a significant number of low peptide AICc scores were further considered for their quantitative correlation using Pearson correlation. Adjusted R^2^ values were assess across peptides for the selected models. Peptides exhibiting strong correlation with the selected model were used for further model refinement with PLS-DA, performed in Metaboanalyst v4.0 [69] using data from half of the subjects selected at random. Features were ranked by VIP scores, with a cutoff at 1.0 [70]. Leave-one-out cross validation was then performed, evaluating accuracy, R^2^ and Q^2^ for robust assessment of the predictive capability for each model without sacrificing sample size [71]. Independently, the input peptide data were scrutinized using non-supervised PCA to rule-out over-fitting of the PLS-DA results [37]. Performance of the model featuring the top-5 ranked VIP peptides was then assessed against a validation cohort of subjects (n = 12) with predicted class probability plots.

## Supplementary Information

**Additional file 1.** Serum peptidomic quantitative dataset and statistical analysis for the highly reproducible 3,532 peptides detected in ≥75% of workers.

**Additional file 2.** Sequence and originating protein data for the 27 identified biomarker model peptides.

## Data Availability

The peptidomic dataset generated and analyzed during the current study is available at the MassIVE repository, MSV000087305, of the ProteomeXchange Consortium, PXD025646 (https://doi.org/doi:10.25345/C5RV4G). Individual metrics and demographics collected on the participating workers are not publicly available as they contain personally identifiable information protected by the U.S. Privacy Act of 1974.
